# Supplementary material for: Chronic Obstructive Pulmonary Disease Is Associated with Worse Oncologic Outcomes in Early-Stage Resected Pancreatic and Periampullary Cancers
Source: Biomedicines. 2023 Jun 10;11(6):1684. doi: 10.3390/biomedicines11061684 (PMC10296111; doi:10.3390/biomedicines11061684)
Supplement: Supplementary file 1 [file biomedicines-11-01684-s001.zip › Supplementary Figures.pdf]

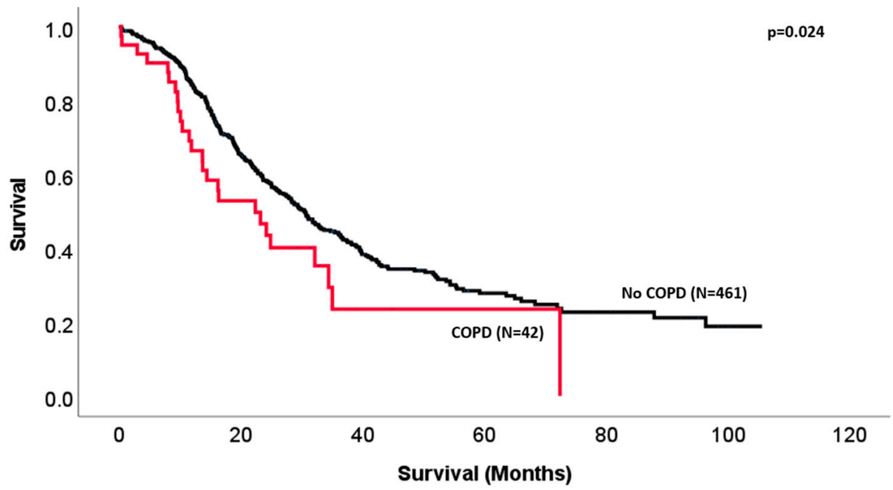

Supplementary Figure S1. Kaplan-Meier overall survival curve of early-stage PDAC and periampullary cancer patients who underwent pancreaticoduodenectomy, comparing survival in patients with chronic obstructive pulmonary disease (COPD) to patients with no medical history of COPD. (Median survival – 22.4±5.4 vs. 30.5±1.8 months, $p=0.024$).
