# Supplementary material for: Chronic Obstructive Pulmonary Disease Is Associated with Worse Oncologic Outcomes in Early-Stage Resected Pancreatic and Periampullary Cancers
Source: Biomedicines. 2023 Jun 10;11(6):1684. doi: 10.3390/biomedicines11061684 (PMC10296111; doi:10.3390/biomedicines11061684)
Supplement: Supplementary file 1 [file biomedicines-11-01684-s001.zip › Supplementary Table.pdf]

Characteristic	Total COPD (n=42)	COPD without steroids (n=34)	COPD with steroids (n=8)	*p*-value
Age, y, median (IQR)	69 (53-86)	70 (66-72)	68 (45-76)	NS
Sex, n (%)				NS
Male	21 (50%)	18 (53%)	3 (37%)	
Female	21 (50%)	16 (47%)	5 (63%)	
BMI (range), kg/m^2	25.5 (19.1-36.6)	25.4 (21.2-29.1)	26.2 (21.9-28.3)	NS
Race, n (%)				NS
White	36 (85.7%)	30 (88.2%)	6 (75.0%)	
Black	5 (11.9%)	3 (8.8%)	2 (25.0%)	
Asian	1 (2.4%)	1 (3.0%)	0 (0%)	
Smoking History, n (%)				
Nonsmoker	6 (14.3%)	2 (5.9%)	1 (12.5%)	NS
Past Smoker	25 (59.5%)	21 (61.8%)	7 (87.5%)	NS
Current Smoker	11 (26.2%)	11 (32.3%)	0 (26.2%)	NS
Neoadjuvant Chemotherapy	4 (9.5%)	4 (11.8%)	0 (0%)	NS
Tumor type, n (%)				
PDAC	38 (90.5%)	31 (91.2%)	7 (87.5%)	NS
Periampullary	4 (9.5%)	3 (8.8%)	1 (12.5%)	NS
Tumor Size, mean (cm)	2.9 ± 1.4	3.2 ± 1.4	2.1 ± 0.8	**0.04**
Lymph Node Involvement, n (%)	29 (69.0%)	25 (67.6%)	4 (50.0%)	NS
Positive Margins, n (%)	6 (14.3%)	6 (17.6%)	0 (0%)	NS
LVI, n (%)	22 (52.4%)	19 (55.9%)	3 (37.5%)	NS
PNI, n (%)	38 (90.5%)	31 (91.2%)	7 (87.5%)	NS
Tumor Staging , n (%)				
T				NS
T1	4 (9.5%)	3 (8.8%)	1 (12.5%)	
T2	15 (35.7%)	10 (29.4%)	5 (62.5%)	
T3	23 (54.8%)	20 (59.8%)	2 (25.0%)	
N				NS
N0	13 (31.0%)	9 (26.5%)	4 (50.0%)	
N1	21 (50.0%)	19 (55.9%)	2 (25.0%)	
N2	8 (19.0%)	6 (17.6%)	2 (25.0%)	

Supplementary Table S1. Tumor and patient characteristics in the COPD subpopulation (n=42). Comparisons between steroid-dependent and non steroid-dependent patients are shown in the right most column. COPD - chronic obstructive pulmonary disease, IQR – interquartile range, PDAC - pancreatic ductal adenocarcinoma, LVI - lymphovascular invasion, PVI - perineural invasion, NS – nonsignificant.

	Factor	HR	*p*-value
Overall survival	Lymphovascular invasion	1.4 (1.0-2.0)	0.03
	Perineural invasion	2.5 (1.2-5.1)	0.013
	Tumor margin positivity	1.6 (1.0-2.4)	0.035
	COPD	1.6 (1.0-2.6)	0.068
Disease-specific survival	Lymphovascular invasion	1.4 (1.0-2.0)	0.03
	Perineural invasion	2.5 (1.2-5.1)	0.013
	Tumor margin positivity	1.6 (1.0-2.4)	0.03
	Neoadjuvant treatment	1.5 (1.1-2.2)	0.02
	COPD	1.5 (0.9-2.4)	0.115

Supplementary Table S2. Cox regression analysis of prognostic factors for overall survival and disease specific survival. Regression model significance $P<0.01$. COPD - chronic obstructive pulmonary disease; HR – hazard ratio.
